# Clinical characteristics and treatment of unilateral allergic fungal rhinosinusitis: a retrospective case series and literature review

**DOI:** 10.3389/falgy.2025.1521574

**Published:** 2025-05-19

**Authors:** Xiaodong Chen, Jiawei Chen, Jian Wang, Min Xu, Tao Xue, Dingjun Zha, Fuquan Chen

**Affiliations:** Department of Otolaryngology-Head and Neck Surgery, Xijing Hospital, Air Force Medical University, Xi’an, China

**Keywords:** rhinosinusitis, fungal allergy, nasal polyps, endoscopic sinus surgery, AFRS

## Abstract

To retrospectively summarize the clinical manifestations, pathological characteristics, and efficacy of unilateral allergic fungal rhinosinusitis (AFRS), we analyzed the clinical data of 23 patients diagnosed with unilateral AFRS in a teaching hospital setting. All the patients showed positive reaction for fungal allergens via the skin prick test or serum-specific IgE test. CT scan showed lesions of the sinuses were unilateral. The average postoperative follow-up time was 72 months. Among the cases, 20 cases were cured, and 3 cases were improved. The VAS score decreased from 8.5 preoperatively to 1.1 postoperatively. The eosinophilic mucin, typical CT findings, and fungal-specific type I hypersensitivity are the three clinical features of AFRS. Endoscopic sinus surgery and oral glucocorticoids are effective treatments for AFRS.

## Introduction

Allergic fungal rhinosinusitis (AFRS) is a unique clinical entity of chronic rhinosinusitis with nasal polyps (CRSwNP), characterized by sticky eosinophilic mucin in expanded sinus cavities, hypersensitivity to fungal elements, and nasal polyposis (1). The Bent and Kuhn criteria remain the most widely accepted diagnostic criteria for AFRS (2). The main evidence includes type I hypersensitivity, nasal polyps, CT scan, non-invasive eosinophilic mucin, and positive fungal staining. Secondary evidence includes asthma, unilateral, bone destruction, fungal culture, Charcot-Leyden crystals, and the increase of serum eosinophilia. The global average incidence of AFRS is 7.8%, with a higher incidence in hot-temperature regions. AFRS often requires surgical intervention combined with long-term corticosteroids and immunotherapy. Recurrence is not uncommon, leading to repeated hospital visits and prolonged medical therapy (3). Although the diagnosis and treatment of AFRS have been reported in the literature, unilateral AFRS has its own characteristics of pathological changes. Some of the patients with unilateral AFRS can develop an erosion of the anterior skull base or orbits, leading to facial or orbital deformities, which needs to be differentiated from neoplasms (4). Moreover, classic Bent-Kuhn criteria for AFRS have met some dilemma and highlight the need to revise the diagnostic criteria for AFRS. The aim of this study was to retrospectively review the clinical data of 23 patients with unilateral AFRS, and further understand the characteristics and treatment strategies of the disease.

## Case series

We retrospectively analyzed the clinical data of 23 patients diagnosed with unilateral AFRS admitted from 2014 to 2023 (Table 1). There were 18 males (78.3%) and five females (21.7%), aged from 29 to 53 years. Lesions of 17 cases were on the left side and six cases were on the right side. The medical history duration was from 3 to 6 months, with an average of 4 months. The duration of postoperative follow-up was from 22 to 130 months, with an average of 72 months.

**Table 1:** Clinical characteristics of the patients.

Characteristic	*n* (%) or median (range)
Total cases	23 (100)
Sex (male/female)	18/5 (78.3/21.7)
Age (years)	38 (29–53)
Affected side (left/right)	17/6 (73.9/26.1)
Medical history duration (months)	4 (3–6)
Affected sinuses	
maxillary sinus	7 (30.4)
ethmoid sinus and sphenoid sinus	7 (30.4)
maxillary sinus, ethmoid sinus, and sphenoid sinus	5 (21.7)
pansinusitis	4 (17.4)
EOS	
EOS count in blood (×10^9^/L)	1.09 (0.77–1.54)
EOS percentage in blood	10.2 (5.4–14.5)
EOS count in mucosa (per HPF)	72 (61–83)
Allergens test finding	
fungi only	8 (34.8)
fungi and other allergens	15 (65.2)
Preoperative VAS	8.5 (7.3–9.7)
Postoperative VAS	1.1 (0.1–2.1)
Efficacy	
controlled	20 (87.0)
partly controlled	3 (13.0)

**Figure 1:**
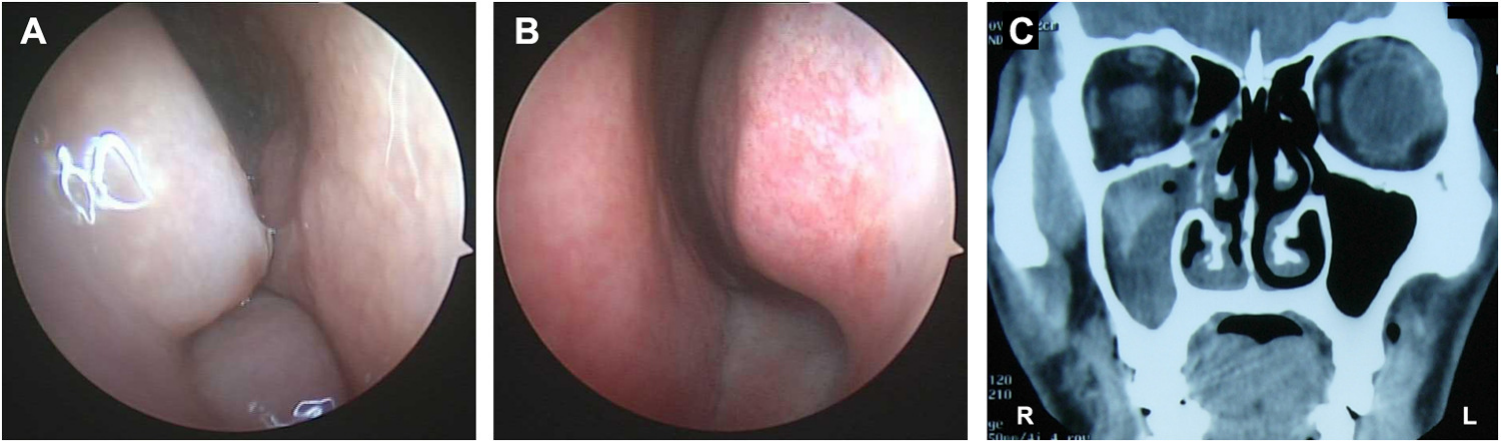
Preoperative nasal endoscopic and sinuses CT finding in AFRS patients. Nasal mucosa on the lesion side was pale and swollen **(A)**, while the contralateral nasal mucosa was normal **(B)**. The affected sinuses were full of irregular, uneven, high-density shadows, which were ground-glass-like, with low peripheral density, and thickened sinus bone **(C)**.

Symptoms include sneezing, nasal obstruction on the affected side, nasal itching, turbid nasal discharge, nasal discharge backflow, dull head pain. Six cases also suffered from decreased sense of smell. Three cases had concomitant asthma. On nasal endoscopy examination, all 23 cases showed polyp or polypoid tissue in the middle nasal passage. Eleven cases showed pale and swollen ipsilateral nasal mucosa, while the contralateral nasal mucosa was normal (Figures 1A,B).

The mean eosinophils count in peripheral blood was 1.09 × 10^9^/L. Among 10 patients undergoing skin prick test, three patients tested positive to simple fungal allergens, and seven tested positive to fungal antigens and other allergens. Among 13 patients undergoing serum-specific IgE test, five patients showed increased IgE level to fungal antigens only, and eight patients showed increased IgE level to fungal antigens and other allergens. CT scan of the sinuses showed that affected sinuses were full of irregular, uneven, ground-glass-like shadows, which were hyperdense areas with low peripheral density, surrounded by thickened inflamed mucosa. Erosive changes of the sinus walls and sinus expansion can also be found (Figure 1C).

**Figure 2:**
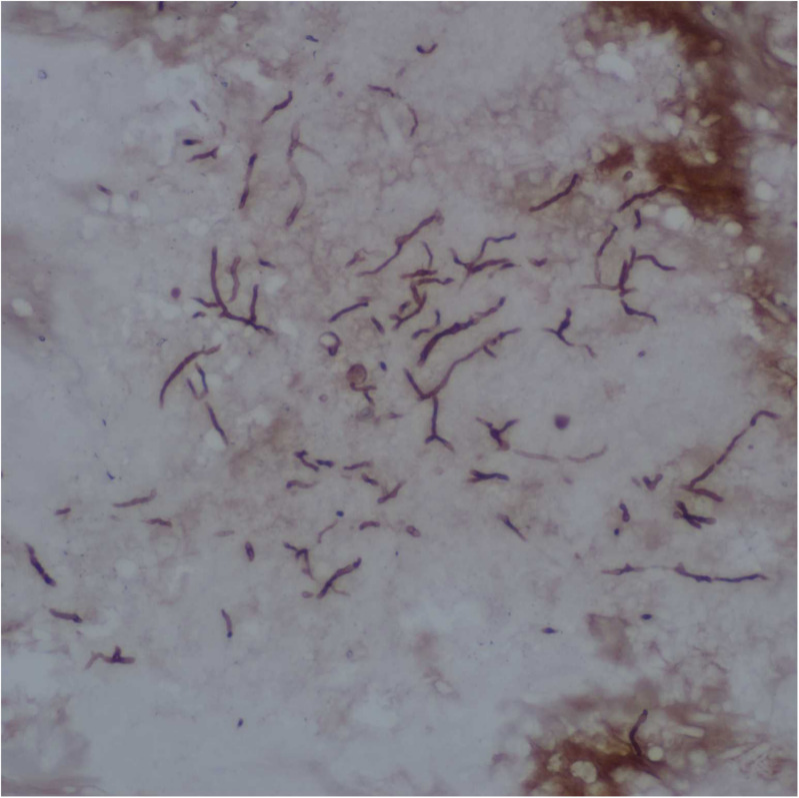
The fungal hyphae and very few fungal spores were stained by silver hexosamine.

**Figure 3:**
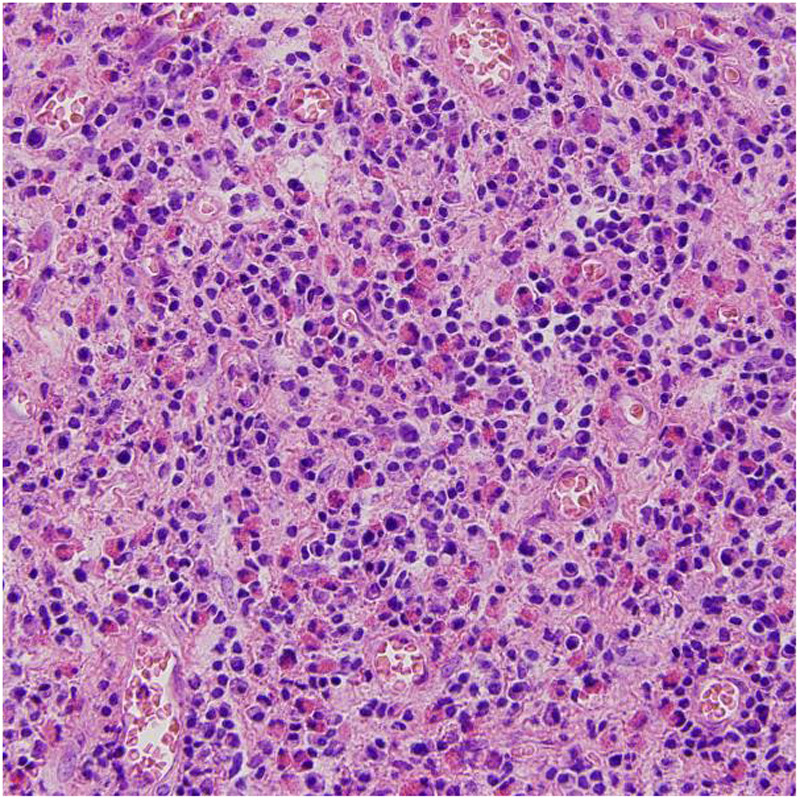
Eosinophils were accumulated in the lamina propria of sinus mucosa.

Intraoperative findings showed a large amount of yellowish-brown mucous accumulated in sinuses. The mucous was extremely viscous, which looked like peanut butter or cheese. Under the microscope, it represented as unstructured eosinophilic mucin with eosinophil accumulation and Charcot-Leyden crystals. The fungal hyphae and very few fungal spores can also be found (Figure 2). The sinus mucosa was congested, swollen, thickened, and some cases showed a paving stone like changes. A large number of eosinophils can be seen in the lamina propria of sinus mucosa (Figure 3), with an average of 72 eosinophils per high power field.

According to the assessment criteria of control of disease in EPOS 2012, the outcomes were categorized as controlled, partly controlled and uncontrolled (5). The clinical evaluation characteristics include nasal blockage, rhinorrhea, facial pain or headache, hyposmia, sleep disorders, abnormal nasal endoscopy findings, and systemic medication needed to control disease. Presence of none of the above characteristic is defined as controlled, presence of only one of the above characteristics is defined as partly controlled, and presence of three or more above characteristics is defined as uncontrolled. Among 23 cases, 20 cases were controlled and three cases were partly controlled in our study. The results of nasal endoscopy showed that no swelling and abnormal secretion occurred in the nasal cavity and sinus three months after the surgery. Postoperative CT examination showed that sinuses of 22 patients recovered to normal. One patient suffered nasal obstruction and frontal sinus pain 16 months after the surgery. CT scan showed ground-glass appearance in the ipsilateral frontal sinus. The average preoperative score of the VAS of disease severity was 8.5, and the average postoperative score was 1.1 (Table 1).

## Discussion

AFRS was first reported in 1976 by Safirstein, who described a patient characterized by extremely thick eosinophilic mucin with fungal hyphae within the sinuses, similar to the pulmonary exudates of the allergic bronchopulmonary aspergillosis (ABPA) (6). At present, AFRS has been widely recognized based on the Bent and Kuhn diagnostic criteria, which emphasize type I hypersensitivity to fungi, nasal polyposis, characteristic imaging signs, eosinophilic mucin with fungal elements, and positive fungal stain of sinus contents. Our findings align with prior studies demonstrating that AFRS predominantly affects young adults, with a higher prevalence in males (7). Unlike some previous reports, our results did not show a correlation between blood eosinophils count and disease recurrence, suggesting that localized eosinophilic mucin may play a more critical role than peripheral eosinophils (8).

AFRS presents several diagnostic hurdles due to its overlapping features with other chronic rhinosinusitis subtypes, particularly eosinophilic chronic rhinosinusitis (eCRS) and fungal ball sinusitis (9). Diagnosis relies on a combination of clinical, radiographic, and histopathologic findings. AFRS often manifests with chronic nasal obstruction, rhinorrhea, and anosmia—symptoms common to many sinonasal disorders. CT images may show hyperattenuating mucus and sinus expansion, but these findings are not pathognomonic and can mimic neoplasms or fungal balls. The histopathologic findings, such as eosinophilic mucin and fungal hyphae on staining, can only be obtained during surgery. All of these factors pose challenges to the early and accurate diagnosis of AFRS. eCRS also presents nasal polyposis and eosinophilic inflammation, but it usually occurs bilaterally. There are a large number of eosinophils in mucosa and mucus of the sinues, but no fungus. Fungal ball sinusitis also occurs unilaterally in most cases but mucin accumulation or allergic reactions are absent (10). CT scan of AFRS is characterized by high-density ground-glass-like shadows in the sinuses, surrounded by soft tissue shadows. Fungal balls appear as multiple spot-like calcifications with uneven density or patch-like high-density shadows (11). In a few cases, AFRS may coexist with the fungal ball. Misdiagnosis of AFRS can lead to inappropriate treatment and additional costs. Reliable serologic or blood-based markers and novel diagnostic methods are needed for AFRS diagnosis.

The pathogenesis of AFRS is still unclear. A previous study identified fungal-specific IgE in the mucin of AFRS, and proposed that AFRS may be a local rather than systemic type I hypersensitivity reaction (12). Tyler et al. studied the molecular characteristics of AFRS and found that the adaptive immune response in AFRS is significantly enhanced compared with non-fungal CRSwNP, and the expression of antibacterial peptides is missing. The lack of antimicrobial peptide may promote the growth of fungal antigens and Th2 immune response (13). Th17 and regulatory T cells (Treg) may also be involved in the pathological process of AFRS, but there have been conflicting reports about their role in AFRS patients of different races (14). The anatomical variations of the nasal cavity and sinuses, such as the vaporization of the middle turbinate and the deflection of the nasal septum, may affect the ventilation and drainage of the sinuses and lead to the accumulation of fungal elements in the sinuses (15).

The mainstay for AFRS treatment remains surgical eradication of eosinophilic mucin followed by corticosteroid therapy. Although surgery cannot control the allergic state of the nasal mucosa, the removal of the lesion helps to establish a good sinus ventilation and drainage, which provides a good basis for drugs to control the allergic reaction of the nasal cavity and sinus mucosa. However, treatment remains challenging because affected sinus cavities may enlarge beyond the reach of sinus surgery instruments, and the difficulty is exacerbated by the extremely sticky nature of the mucus (1). Extended courses of oral corticosteroids postoperatively were recommended in the past. However, doses and duration-dependent adverse effects, such as hyperglycemia, obesity and osteoporosis, has been appreciated and limited long-term use of oral steroids. Recently, topical corticosteroids are highlighted as they have few systemic effects. Some exhalation delivery system has shown significant improvement in delivery and symptoms in postoperative patients with open sinuses of CRS patient (16).

All the cases in the present study underwent surgery and local glucocorticoids treatment. Those with obvious postoperative swelling of the sinus mucosa were treated with short-term systemic glucocorticoids. Since the fungus is not a pathogen of AFRS, systemic antifungal drugs are generally not recommended (17). Recently, anti-IgE targeted biological agents have been report to achieved good results in the treatment of AFRS (18). It can help to reduce the dependence on glucocorticoids and become a new choice for patients with poor response to conventional therapy.

Our results reinforce prior evidence that functional endoscopic sinus surgery significantly improves symptom control and reduces recurrence rates. However, our study has several limitations. The time span of our case records is relatively large, some data may be incomplete. For example, medical history and follow-up details may not be uniformly documented. Additionally, our study lacked an investigation of postoperative immunological parameters, which can be used as objective indicators for efficacy evaluation. Future studies should focus on exploration of biomarkers (e.g., fungal-specific IgE, IL-5, eosinophil-derived neurotoxin) to predict disease severity and treatment response. Prospective multicenter studies are needed to validate diagnostic criteria, and assess the efficacy of biologics in reducing corticosteroid dependence, particularly in refractory cases.

## Conclusion

Eosinophilic mucin, typical CT findings, and fungal-specific type I hypersensitivity are the three clinical features of AFRS. Systemic and local allergic reaction may be associated with the onset of unilateral AFRS. Endoscopic sinus surgery and oral glucocorticoids are effective treatments for AFRS.

## Data Availability

The original contributions presented in the study are included in the article/Supplementary Material, further inquiries can be directed to the corresponding authors.
